# Evaluation of the proliferation markers Ki-67/MIB-1, mitosin, survivin, pHH3, and DNA topoisomerase IIα in human anaplastic astrocytomas - an immunohistochemical study

**DOI:** 10.1186/1746-1596-6-43

**Published:** 2011-05-24

**Authors:** Andreas H Habberstad, Sasha Gulati, Sverre H Torp

**Affiliations:** 1 Department of Laboratory Medicine, Children's and Women's Health, Norwegian University of Science and Technology, Trondheim, Norway; 2 Department of Neurosurgery, St. Olav's University Hospital, Trondheim, Norway; 3 Department of Pathology and Medical Genetics, St. Olav's University Hospital, Trondheim, Norway

## Abstract

**Background:**

Histological malignancy grading of astrocytomas can be challenging despite criteria given by the World Health Organisation (WHO). Grading is fundamental for optimal prognostication and treatment, and additional biomarkers are needed to support the histopathological diagnosis. Estimation of proliferative activity has gained much enthusiasm, and the present study was designed to evaluate and compare novel immunohistochemical proliferative markers in human anaplastic astrocytomas.

**Methods:**

Proliferative activity was determined in twenty-seven cases with antibodies reactive against the Ki-67 antigen, mitosin, survivin, pHH3, and DNA topoisomerase IIα, and they were mutually compared as well as related to mitotic activity.

**Results:**

The markers correlated well with each other, but poorly with mitoses, probably because of small and squeezed tumour samples, in which identification of mitoses can be difficult. Positive association to overall survival was observed as well.

**Conclusions:**

Our data show that these markers may assist significantly in the evaluation of proliferative activity in anaplastic astrocytomas and even have prognostic value.

## Introduction

Diffuse astrocytomas are the most common primary malignant brain tumours in humans. They are characterized by widespread distribution throughout CNS, diffuse and infiltrative growth pattern, and inherent trend to undergo malignant transformation. In general the prognosis is poor despite progress in tumour imaging and treatment.

Histopathologic diagnosis is essential for optimal prognostication and treatment. According to World Health Organization (WHO), diffuse astrocytomas can be divided into diffuse astrocytoma grade II, anaplastic astrocytoma grade III, and glioblastoma grade IV [1]. Distinction between different tumour grades can be challenging, and limited tumour material is often provided to the pathologist. The number of mitoses is of paramount importance, but can be hard to identify in haematoxylin and eosin (H&E)-stained sections [2].

Since proliferative activity is a reliable method to assess tumour biology, there has been continuous research to find such biological markers. Commonly used is the monoclonal antibody Ki-67/MIB-1 which has proven prognostic and diagnostic power in astrocytic tumours [3,4]. Nevertheless, the application of this antibody is hampered by lack of standardization of the immunohistochemical procedures, significant interlaboratory variability, and considerable overlap between the different malignancy groups [3,4].

New antibodies reactive against proliferation-associated antigens have been launched and shown to correlate with tumour grade, mitoses, and Ki-67/MIB-1 [5-12]. The core histone protein H3 constitutes a major part of the chromatin and is phosphorylated during mitosis [13,14]. There are reports on the phosphorylated form of histone H3 (pHH3) at serine 10 and its potential clinical role and prognostic value in astrocytomas [5,6].

Another marker is survivin, a member of the inhibitor-of-apoptosis-family, which promotes survival of tumour cells [7,15]. It is commonly expressed in embryonic and neoplastic tissues and barely expressed in normal cells [16]. There is limited experience with survivin immunostaining and prognosis in anaplastic astrocytomas, and conflicting data exist in glioblastomas [8,17,18]. Even the significance of the subcellular localization of this protein appears uncertain [6,17-19]. Further, survivin may promote radiation resistance in glioblastomas [20,21].

Nuclear DNA topoisomerase IIα (TIIα) serves as an essential enzyme with important function in DNA topology, repair, and replication, and proliferative signals may upregulate the gene expression [22,23]. TIIα immunostaining in high grade astrocytomas has been shown to represent a reliable proliferation marker and to provide valuable prognostic information [8-12,24].

Mitosin, also called p330d/CENP-F, is linked with the centromere/kinetochore complex and is expressed during the active phases of the cell cycle with a maximum in G2 and M [25,26]. Increased expression is associated with malignancy grade and survival of astrocytomas [12], however, there are few studies to support this finding.

It appears that current proliferation markers to varying degree hold prognostic significance in human astrocytic tumours, however, the experience in anaplastic astrocytomas is limited. The goal of the present study was to evaluate and compare these novel proliferation markers and consider their prognostic value in a series of anaplastic astrocytomas.

## Materials and methods

This study is an extension of a recently published study [27]. A total number of 27 patients with supratentorial anaplastic astrocytomas operated at the Department of Neurosurgery, St. Olav's University Hospital, Trondheim, Norway, in the time period 1998-2006, were included. The extent of tumour resection was determined by postoperative MRI scans. Surgical resection was defined as gross total resection, partial resection, or biopsy. The clinical data were obtained from electronic medical records and included age, sex, symptoms at presentation, tumour localization, treatment modalities, and postoperative survival. Preoperative Karnofsky performance status (KPS) score was retrospectively determined from a routine neurological examination from patient admittance.

The tumour samples consisted of both formalin-fixed and paraffin-embedded cryosections and unfrozen tissue. All haematoxylin and eosin (H&E) stained sections were reviewed by an experienced neuropathologist (SHT), and tumour grading was based on the latest WHO scheme [1]. The commercial antibodies used are shown in Table 1. Four μm thick sections with representative tumour tissue were incubated with primary antibodies after quenching of endogenous peroxidase activity with 3% hydrogen peroxyde and antigen retrieval by pressure cooking. The immunostaining was carried out on a DAKO Autostainer (Dako, Glostrup, Denmark). Visualization of immunoreactivity was performed with DAKO EnVision system with diaminobenzidin as chromogene. Sections were counterstained with haematoxylin. Positive controls were included in each staining run (human tonsils).

**Table 1:** Antibodies and immunohistochemical procedures

Antibody	Source	Type	Clone	Positive control	Dilution	Incubation temperature	Incubation time
**Ki-67/MIB-1**	Dako, Glostrup, DK	Monoclonal	Ki-67 antigen	Tonsil	1:100	Room temperature	40 min
**PHH3**	Upstate Biotechnology. Millipore, Billerica, USA	Polyclonal	H3 (Ser10)	Tonsil	1:2000	Room temperature	60 min
**Survivin**	Abcam Products, Cambridge, MA, USA	Monoclonal	EP2880Y	Tonsil	1:250	Room temperature	40 min
**Mitosin**	Novus Biologicals, LTD, Cambridge, UK	Monoclonal	14C10/1D8	Tonsil	1:500	Room temperature	40 min
**DNA topoisomerase IIα**	Dako, Glostrup, DK	Monoclonal	KiS1	Tonsil	1:100	Room temperature	40 min

For the microscopical analyses a Nikon Eclipse 80i was used. All immunostained sections were scanned for the most densely labelled areas, and calculations were done with the 40x objective, termed microscopic high power fields (HPF), using an ocular grid (0.058 mm^2^).

Mitotic activity was defined as the number of mitoses in ten consecutive HPFs, whereas mitotic index was calculated as the percentage of mitoses using the ocular grid. In several tumour samples mitotic counting was difficult or impossible due to small and squeezed biopsies. Cells with positive pHH3 staining were recorded in the same way as mitoses, including only positively stained nuclei with chromatin changes equivalent to mitotic division.

**Figure 1:**
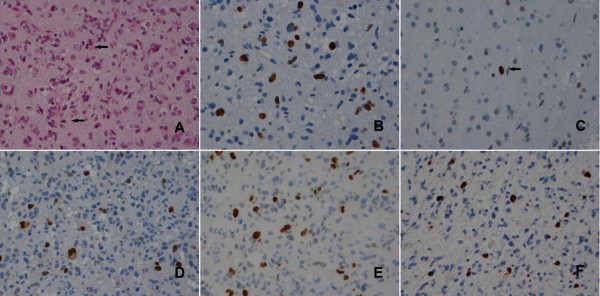
**Proliferation markers in anaplastic astrocytomas**. Mitotic figures (arrows) in H&E-stained sections (A). Nuclear immunoreactivity of Ki-67/MIB-1(B), pHH3 stained mitotic figure (arrow) (C), survivin (D), TIIα (E), and mitosin (F).

Antibodies against Ki-67, pHH3, survivin, mitosin, and TIIα displayed immunoreactive tumour cell nuclei (Figure 1). Only distinctly stained nuclei were counted. The labelling index (LI) was determined by counting at least 1000 tumour cells in the ocular grid in one HPF. If this number of tumour cells could not be obtained, three HPFs were examined. The proliferative index was defined as the percentage of immunoreactive tumour cells out of the total number of cells. This part of the study was executed blindly from any clinical information, and the data were evaluated by spot tests of the results from two independent scientists (SHH and AHH). Regarding survivin immunostaining, the applied monoclonal antibody was reactive against both cytoplasmic and nuclear located protein and we recorded only the latter.

Statistical analyses were assessed using SPSS version 17.0 (SPSS Inc., Chicago, IL) Correlations between variables were identified employing the Spearman's rank correlation (Spearman's rho), survival data by using Kaplan-Meier curves, log rank-, and COX regression test. Cut points for the different variables were set according to the literature or at the median. The p value, from which statistical significance was assumed, was set to p < 0.05.

The study was approved by the Regional Committee for Medical Research Ethics and protocols followed the guidelines by the Helsinki Declaration.

## Results

### General

Twenty-seven patients (10 women and 17 men; median age, 49 yr; age range, 28-78 yr; median KPS, 70; KPS range, 50-90) with anaplastic astrocytomas were included in the study. Clinical data are presented in Table 2. Four of the patients had previously undergone brain surgery for diffuse astrocytoma (WHO grade II).

**Table 2:** Patient characteristics

**Case no**.	Age (years)/Sex	Prior brain tumour	KPS^1^	Resection grade	Radiotherapy	Postoperative survival (months)
1	28/F^2^	No	90	GTR^3^	1.8 Gy × 30	54.6
2	32/F	No	70	Partial	2.0 Gy × 30	16.0
3	34/F	No	80	Partial	1.8 Gy × 30	29.0
4	34/F	No	80	Partial	1.8 Gy × 30	94.4+^4^
5	36/M^5^	No	70	Partial	3.0 Gy × 13	11.1
6	37/M	No	80	GTR	No	53.8+
7	38/M	No	80	Partial	2.0 Gy × 30	34.8+
8	39/F	No	70	GTR	N.D.^6^	N.D.
9	42/M	No	70	GTR	1.8 Gy × 30	92.3+
10	44/F	No	80	GTR	No	58.9+
11	44/M	Low-grade glioma	90	Partial	No	40.9
12	44/M	Low-grade glioma	80	Partial	1.8 Gy × 30	29.0+
13	47/M	Low-grade glioma	90	GTR	No	88.8+
14	49/M	No	50	Biopsy	No	0.1
15	49/M	No	70	Partial	2.0 Gy × 30	30.2+
16	50/M	Low-grade glioma	80	Partial	1.8 Gy × 30	23.4
17	53/M	No	70	Partial	1.8 Gy × 30	24.3
18	56/F	No	70	Partial	2.0 Gy × 30	17.6
19	57/F	No	70	Partial	1.8 Gy × 30	5.5
20	57/F	No	80	Partial	1.8 Gy × 30	62.9+
21	57/M	No	80	Partial	1.8 Gy × 30	16.4
22	58/M	No	80	Partial	1.8 Gy × 30	28.2
23	64/M	No	70	GTR	1.8 Gy × 30	75.0+
24	71/M	No	50	Biopsy	3.0 Gy × 13	10.1
25	72/M	No	60	Biopsy	1.8 Gy × 30	10.9
26	78/F	No	50	Biopsy	3.0 Gy × 13	9.7
27	78/M	No	50	Biopsy	No	1.8

^1^KPS = Karnofsky Performance Status, ^2^F = Female, ^3^GTR = Gross total resection, ^4^(+) denotes that the patient is still alive, ^5^M = Male, ^6^N.D. = No data.

### Proliferative markers

Immunohistochemical data and estimates for mean values for the proliferative markers are shown in Table 3. Correlation computed by the Spearman's rho, showed significant correlation between most of the proliferative markers (Table 4). Only survivin correlated significant to mitotic activity. The strongest correlations were seen between survivin and the pHH3 index (*r_s _*= 0.779, p < 0.001), survivin and Ki-67/MIB-1 (*r_s _*= 0.666, p < 0.001), survivin and mitosin (*r_s_*= 0.627, p = 0.001), TIIα and pHH3 mitoses/10HPF (*r_s _*= 0.611, p = 0.001), survivin and TIIα (*r_s_*= 0.552, p = 0.003), and between mitosin and Ki-67/MIB-1 (*r_s _*= 0.558, p = 0.005).

**Table 3:** Results of the immunohistochemical analyses of the proliferation markers

**Case no**.	Mitoses/10HPF	Mitotic index	Ki-67 /MIB-1	PHH3 mitoses /10HPF	PHH3 index	TIIα	Survivin	Mitosin
1	N.D.^1^	N.D.	6.8	1	0.5	1.8	2	2.4
2	0	0	3.5	1	1.7	2.5	2.7	8.5
3	15	0.9	6.8	19	0.9	10.8	5.3	3.4
4	1	0.6	9.2	18	0.9	7.9	7.5	8.6
5	0	0	19.0	6	2.2	0	5.8	3.6
6	2	0.4	9.2	2	0.6	2	3.4	3.1
7	0	0	1.0	0	0.0	0.8	0.3	0
8	1	0.6	7.9	1	0.8	2.7	3.3	3.5
9	0	0	5.0	1	0.2	0	2.4	2.5
10	0	0	2.1	3	0.6	0	1.9	1.8
11	N.D.	N.D.	6.4	10	1.3	25	3.7	1.5
12	0	0	4.2	4	1.0	4	2.7	1.9
13	N.D.	N.D.	4.4	4	0.6	1.8	1.2	1.1
14	N.D.	N.D.	8.5	8	0.8	5	2.6	3.4
15	6	0.5	22.2	9	1.1	8.2	6	3.8
16	N.D.	N.D.	8.6	1	0.5	6.1	2.5	8
17	1	0.7	N.D.	N.D.	N.D.	4.5	3.8	2.9
18	N.D.	N.D.	15.0	5	1.9	6	4.4	2.7
19	0	0	20.0	2	0.4	5.4	2.3	1.4
20	N.D.	N.D.	3.2	1	0.1	1.4	1.5	N.D.
21	N.D.	N.D.	16.4	30	1.1	6.9	3.5	4.5
22	2	0.5	4.0	5	0.8	3.7	3.5	1.2
23	1	0.8	12.9	15	2.0	0	6.1	2.7
24	N.D.	N.D.	4.9	16	1.5	2	2	2
25	0	0	16.5	24	1.7	11	8.9	5.1
26	0	0	10.2	19	0.6	6.3	2.6	N.D.
27	13	0.7	21.0	37	2.7	13.5	9.6	9.5
								
Median	0.5	0.2	8.2	5	0.8	4	3.3	2.9
Minimum	0	0	1	0	0	0	0.3	0
Maximum	15	0.9	22.2	37	2.7	25	9.6	9.5
								
								
Mean	2.3	0.3	9.6	9.3	1.0	5.2	3.8	3.6
Std. deviation	4.5	0.3	6.3	10.0	0.7	5.4	2.3	2.5
Variance	20.2	0.1	39.6	100.2	0.5	28.9	5.2	6.4

^1^N.D. = No Data

**Table 4:** Relationships between the proliferation markers (Spearman's rank correlation)

		Mitoses/10HPF						
					
Mitotic index	*r_s_*	.864^1^	Mitotic index					
	p value	0.000						
				
Ki-67/MIB-1	*r_s_*	.292	.238	Ki-67/MIB-1				
	p value	0.255	0.358					
			
PHH3 mitoses /10HPF	*r_s_*	.402	.404	.543	PHH3 mitoses/10HPF			
	p value	0.110	0.108	0.015				
		
PHH3 index	*r_s_*	.320	.361	.470	.661	PHH3 index		
	p value	0.211	0.154	0.015	0.000			
	
TIIα	*r_s_*	.430	.303	.479	.611	.352	TIIα	
	p value	0.075	0.222	0.013	0.001	0.078		
Survivin	*r_s_*	**.582**	**.588**	**.666**	**.672**	**.779**	**.552**	Survivin
	p value	**0.011**	**0.010**	**0.000**	**0.000**	**0.000**	**0.003**	

Mitosin	*r_s_*	.312	.261	**.558**	.372	**.475**	**.443**	**.627**
	p value	0.223	0.311	**0.005**	0.074	**0.019**	**0.027**	**0.001**

^1 ^p value < 0,05 is highlighted

### Survival

The median survival was 2.4 years (95% CI, 8-48 months). The one-year survival was 73%. Univariate COX analyses with overall survival as the dependent variable, demonstrated significant correlation to KPS (p < 0.001), tumour surgery (p < 0.001), age (p = 0.016), pHH3 index (p = 0.018), and Ki-67/MIB-1 (p = 0.031). Gross total resection and higher KPS independently correlated with overall survival (p = 0.026 and p = 0.001 respectively) in a multivariate COX analysis.

**Figure 2:**
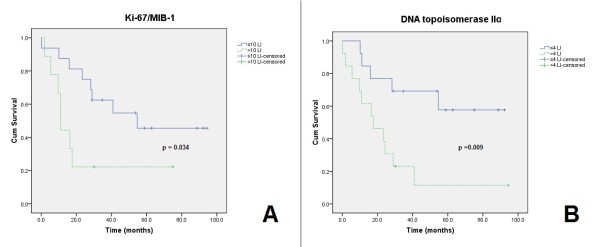
**Kaplan-Meier survival curves**. KI-67/MIB-1 (mean OS = 58 vs. 25 months) (A). TIIα (mean OS = 65 vs. 26 months) (B).

Log-rank tests and Kaplan-Meier curves were computed for the cut off values of each proliferation marker. Overall survival (OS) was the dependent variable. Ki-67/MIB-1 ≤10 versus >10 (mean OS = 58 vs. 25 months, p = 0.034) and TIIα ≤4 versus >4 (mean OS = 65 vs. 26 months, p = 0.009) resulted in significant differences (Figure 2), while pHH3 index ≤0.8 versus >0.8 showed a trend (p = 0.089). The other proliferative markers did not reach statistical significance.

## Discussion

In astrocytomas proliferative activity has been positively correlated to tumour grade and prognosis. Small biopsies and intricate histology make diagnosing difficult, and reliable biological markers are highly needed. In this immunohistochemical study, we demonstrated positive correlation between the proliferation markers Ki-67/MIB-1, mitosin, survivin, pHH3, and DNA topoisomerase IIα. Furthermore, Ki67/MIB-1 and pHH3 indicated poorer survival in univariate analyses.

Mitotic activity is fundamental in the histopathologic grading of human astrocytomas. Identification of mitotic figures is hampered by several factors including squeezed cells in stereotactic biopsies, distortion and similarities to chromatin changes in apoptotic and pycnotic cells [2]. We experienced such elements, particularly in small biopsies and in paraffin sections prior used in frozen sections diagnostics. In contrast to others [5,6,28,29] we did not find positive correlations between mitoses and most of the proliferative markers, probably because of the above mentioned factors. Hence, this observation suggests that antibodies against proliferation-associated antigens are useful to obtain an optimal profile of the proliferative activity, especially in small brain tumour samples.

Ki-67/MIB-1 immunostaining worked well and yielded credible results in our study. The LIs displayed a wide range of values that overlap with indices in both grade II and grade IV astrocytomas [3], and is regarded as the main reason for Ki-67/MIB-1 not being included in the routine histopathological diagnosis of astrocytic tumours [1]. Thus, this marker should not be used alone, but in combination with established histopathological criteria of malignancy.

Proposals for clinical threshold values have been suggested [3] without a consensus being reached. Using a value of 10% [3,7] a significant impact on survival was found, verifying this as a reasonable threshold.

pHH3 is a novel promising proliferation marker in tumour pathology, however the experience in astrocytomas is limited. In our hands, this antibody provided reliable immunostaining with distinct nuclear positivity in cells with mitotic morphology. Positive correlations with the other proliferative markers were found except mitoses. This discrepancy may be due to the problem to identify mitotic figures in squeezed and small biopsies. Comparison between the number of mitoses and pHH3 positive nuclei, revealed the latter to be higher, in accordance with being a more sensitive marker for mitoses [5,14,29,30]. A major drawback of the pHH3 immunostaining seems to be positivity in non-mitotic cells [14]. This, together with the subjective determination of mitotic morphology, may lead to misinterpretation. We demonstrated that higher indices were associated with poorer survival in univariate analyses in accordance with others [5]. For this reason pHH3 stands out as a reliable biomarker, however, larger studies are necessary to further elucidate this observation.

Survivin immunoreactivity was located both in the cytoplasm and in the nucleus, as described by others [6,8,18]. The nuclear positivity was in contrast to the cytoplasmatic staining, easily detectable and distinct. As the localization of survivin compartments in the nucleus is correlated to cell division and prognosis [6,17,19], only the nuclear positivity was included. Survivin expression showed significant positive correlation with all of the proliferation markers, suggesting that nuclear survivin positivity can be a reliable proliferation marker in astrocytic tumours. Our data demonstrated no correlation between survivin expression and survival. The literature is contradictory in this matter [8,17-19], and larger studies are needed to clarify this topic. The effect of survivin on radiotherapy resistance is intriguing. If, however, radiotherapy effects depend on the presence or not of survivin, the implication in anaplastic astrocytomas can be limited, as all of the tumours in our material displayed positively stained cells.

The results from our TIIα immunostaining correlated well to the other proliferation markers, in agreement with previous reports [8,11,24]. The number of positively stained nuclei was easily calculated, and was in general of a lower value than the values of Ki-67/MIB-1, as seen in previous studies [11,24]. This result could be explained by the different protein expression throughout the cell-cycle. TIIα positive cells in G0- and G1 phase have been reported [9], and may represent a drawback of this marker. Using the median of 4% as a cut-point, TIIα was significantly associated with survival in our material. This is in accordance with others using similar cut-points [9] and suggests that TIIα could be of prognostic value in astrocytic tumours.

Mitosin also displayed significant correlations to all of the proliferative markers, which is in accordance with studies on other malignancies [26]. Immunoreactive nuclei were easily identified and can be explained by the mitosin expression in S-, G2-, and M phase, and its rapid degradation [25,26]. Our study could not correlate mitosin expression significantly to survival.

Due to the small number of cases, the survival analyses have been carefully interpreted. However, univariate analyses have gathered sense of which markers that could be further investigated in well-designed studies for prognostic relevance.

The caveats of immunohistochemical analyses include different antibodies, different antigens, background staining, and inhomogeneous staining that can contribute to interlaboratory and interobserver variations. Wrong assumptions can also be made as the knowledge of the novel markers is limited. For instance, protein overexpression in tumour cells can represent genetic aneuploidity, mutated genes or increased transcription factors. Additionally, the functions of a proliferative marker may not exclusively be related to the cell cycle, as was the case for the proliferating cell nuclear antigen (PCNA) [31].

Variations in immunoreactivity may be due to different expression during the cell cycle. The recorded wide range of values represents a major drawback of such markers as one will experience considerable overlap between malignancy groups. Thus, one should consider the possibility to introduce a panel of proliferation markers to identify more aggressive astrocytomas.

## Conclusion

In conclusion, immunohistochemical determination of proliferative activity in anaplastic astrocytomas using antibodies against Ki-67 antigen, survivin, mitosin, pHH3 and DNA topoisomerase IIα, may assist in the histopathological diagnosis, especially because mitoses can be hard to detect. The prognostic value requires further investigation.

## Competing interests

The authors declare that they have no competing interests.

## Authors' contributions

AHH participated in the immunohistochemical and the statistical analyses, and drafted the manuscript. SG collected clinical information, performed statistical analyses and participated in the design of the study and to draft the manuscript. SHT conceived of the study and participated in its design, coordination and immunohistochemical analyses and helped to draft the manuscript. All authors read and approved the final manuscript.
